# The impact of PTSD symptoms on post-disaster consumption: tertiary victims of the 2023 Kahramanmaraş earthquakes in Türkiye

**DOI:** 10.3389/fpubh.2026.1703071

**Published:** 2026-02-24

**Authors:** İbrahim Kırcova, Munise Hayrun Sağlam, Ahmet Can Şenlik, Doğan Mert Akdemir, Ebru Enginkaya

**Affiliations:** 1 Department of Business Administration, Yıldız Technical University, Istanbul, Türkiye; 2 Department of Aviation Management, Istanbul Gelişim University, Istanbul, Türkiye; 3 Department of International Trade, Istanbul Ticaret University, Istanbul, Türkiye

**Keywords:** death anxiety, intolerance of uncertainty, Kahramanmaraş earthquake, media exposure, post-disaster consumer behavior, post-traumatic stress disorder (PTSD), search for meaning

## Abstract

**Introduction:**

The February 2023 Kahramanmaraş earthquakes reshaped everyday life well beyond the impact zone; however, how disaster-linked psychological states influence psychosocial wellbeing and everyday behaviors, including consumer responses among tertiary victims (geographically distant yet psychologically affected), remains underexplored.

**Methods:**

We employ an explanatory sequential mixed-methods design (QUAN → QUAL): a cross-sectional survey of Istanbul adults (*N* = 350) is modeled using PLS-SEM, followed by 24 semi-structured interviews and reflexive thematic analysis, integrated via joint displays.

**Results:**

Quantitatively, PTSD relates to post-disaster wellbeing and consumption directly and indirectly through death anxiety (DA), intolerance of uncertainty (IUS), and search for meaning (MLQ-S); perceived media pressure (ME) attenuates these translations on average. Qualitatively, participants described securing basics and redundant backups as control-restoration and “emotional insurance” to preserve safety, small indulgences as low-guilt self-care that supports emotional health, media as a double-edged influence (unregulated viewing amplifies anxiety; deliberate curation dampens it), and purposeful, value-aligned purchases as identity repair and resilience-building.

**Discussion:**

The findings extend terror-management, control-restoration, and meaning-making accounts to vicarious-trauma contexts and identify media regulation as a key boundary condition. Practically, they support public health risk communication that normalizes selective exposure, ethical preparedness that restores agency and wellbeing without excess, and interventions that channel recovery toward responsible self-care and value-aligned choices.

## 1 Introduction

On February 6, 2023, two powerful earthquakes (Mw 7.7 and 7.6) struck the Turkish districts of Pazarcık and Elbistan, followed by a Mw 6.4 event near Yayladağ on February 20 (1). These earthquakes represent the most severe disaster in Türkiye’s recent history, destroying infrastructure across 11 provinces and claiming over 48,000 lives. Beyond immediate destruction, such large-scale disasters trigger profound psychosocial and public health consequences, reshaping everyday life, coping strategies, and decision-making well beyond the epicenter.

Disaster exposure is closely linked to psychological morbidity, constituting a significant public health concern. Post-traumatic stress disorder (PTSD), characterized by intrusive recollections, avoidance, negative alterations in cognition and mood, and hyperarousal, is among the most frequently observed outcomes after catastrophic events (2, 3). Elevated rates of stress, anxiety, and PTSD have been documented following mass trauma, including the September 11 terrorist attacks (4). Importantly, such reactions are not limited to direct survivors. Disasters function as “collective stress” events that affect geographically distant populations through symbolic threats and mediated exposure (3, 5, 6). Evidence from Japan’s Tōhoku earthquake shows that even those far from the epicenter can develop PTSD symptoms through vicarious exposure (7), with severity shaped by emotional proximity to victims and concern for loved ones.

PTSD-related distress can also spill over into daily life management and coping behaviors, including consumption patterns, with implications for well-being and preparedness. Individuals often turn to buying to regain control, redirect attention, or regulate negative emotions (8, 9). While prior studies have linked disaster-related trauma to compulsive or replacement purchasing (10, 11), most research has focused on vice goods (alcohol, tobacco, narcotics), leaving broader everyday domains underexplored (12). In Türkiye, heightened scrutiny of building code compliance and repeated amnesties has amplified perceived structural risk, steering even tertiary victims toward preparedness goods, earthquake-resilient products, and insurance uptake (DASK) (13). These dynamics reveal how trauma intersects with everyday behaviors in ways directly relevant to psychosocial health and disaster resilience.

This study addresses that gap by focusing on tertiary victims—individuals geographically distant from the disaster area yet psychologically affected through emotional or mediated proximity (5). Specifically, we examine how PTSD symptoms among tertiary victims relate to post-disaster consumption behaviors (CBDD) through three theoretically grounded mediators: DA, IUS, and MLQ-S. We also test whether perceived media pressure moderates these pathways, given the central role of media in sustaining or dampening disaster salience. An explanatory sequential mixed-methods design (QUAN → QUAL) is employed: a structural model estimated via PLS-SEM, followed by semi-structured interviews that contextualize the mechanisms, boundary conditions, and lived interpretations of the quantitative links.

Despite Türkiye’s high seismic risk and the persistent salience of earthquakes, the psychosocial and health-related implications of post-disaster consumption among tertiary victims remain underexplored. In this study, tertiary victims are defined as residents of İstanbul who were not directly affected by the February 2023 Kahramanmaraş earthquakes but were repeatedly exposed to their consequences through media coverage and social networks.

Existing Turkish-context scholarship has examined disaster response management (14), brand communication (15), governance and compliance (13), and humanitarian logistics (16). However, it has not systematically linked PTSD-related states to everyday behaviors with implications for well-being. By focusing on this population, the present study expands our understanding of how disaster-related psychological states relate to coping logics such as precautionary necessity purchasing, hedonic self-care, and value-aligned spending, and how media influences these pathways. In doing so, it contributes to both marketing and public health scholarship by clarifying how trauma-related distress affects psychosocial adjustment, well-being, and resilience at a societal level.

## 2 Theoretical framework

Disasters represent multidimensional stressors that affect not only immediate survivors but also geographically distant populations who experience trauma vicariously through symbolic threats and media exposure (3, 7). Understanding how such psychological states shape everyday behaviors requires an integrative theoretical lens. This study draws on Terror Management Theory (TMT) as its primary framework, complemented by Stress and Coping Theory (SCT) (17) and Conservation of Resources Theory (CRT) (18), to capture the complex interplay between mortality salience, coping, and consumption as psychosocial adaptation. Building on these perspectives, we draw on the notion of tertiary traumatization to capture trauma related processes among individuals who are not directly exposed to a disaster but are repeatedly confronted with its consequences through media coverage, interpersonal narratives, and institutional discourse. In contemporary disaster ecologies, high intensity and prolonged media exposure can evoke intrusive imagery, hyperarousal, and anticipatory fear even among geographically distant populations, particularly when they live under similar structural risks such as the widely recognized seismic threat in İstanbul. In such contexts, individuals may internalize a persistent sense of vulnerability and symbolic proximity to catastrophe, which can manifest in posttraumatic stress symptoms despite the absence of direct physical impact.

These tertiary trauma processes are closely intertwined with coping efforts that are enacted through consumption. Some individuals respond to heightened threat and loss of predictability by engaging in preparedness oriented and risk management consumption, such as investing in safety products, insurance, or emergency supplies, while others turn to hedonic or self soothing consumption to regulate distress. At the same time, perceptions of uncontrollability and institutional failure in disaster preparedness and response can foster feelings of learned helplessness for a subset of individuals, reinforcing passivity and withdrawal. For others, however, the same appraisals may trigger compensatory attempts to regain a sense of control and moral agency through resource accumulation, solidarity oriented spending, and value aligned consumption choices. This duality provides an important conceptual bridge between trauma related symptomatology and the heterogeneous consumption patterns observed in our study. These theories provide a multi-level explanation of how PTSD symptoms among tertiary victims translate into consumption patterns. TMT explains how mortality salience fuels existential defenses, which are manifested in necessity and hedonic purchases. SCT situates these behaviors as part of a broader coping repertoire that influences psychosocial well-being. COR theory highlights the resource logic underlying preparedness and redundancy, framing consumption as a means of protecting and replenishing threatened resources. By integrating these perspectives, the present study conceptualizes CBDD as not merely economic activity but as a health-relevant coping process that reflects attempts to restore agency, mitigate anxiety, and preserve well-being in the aftermath of collective traumafield.

### 2.1 Terror management theory and mortality salience

TMT posits that awareness of mortality generates existential anxiety, which individuals manage through cultural worldviews, self-esteem, and symbolic practices that provide a sense of meaning and continuity (19, 20). Traumatic events such as earthquakes make death salient, triggering compensatory responses aimed at reducing fear (21). Empirical research shows that mortality salience can increase both precautionary behaviors (e.g., preparedness purchasing) and hedonic consumption as proximal or distal defenses (10). In the context of tertiary victims, consumer responses such as stockpiling, low-guilt indulgences, or value-aligned purchases can be understood as coping strategies that buffer existential threats and restore psychological stability.

### 2.2 Stress and coping theory

Lazarus and Folkman’s (17) transactional model of stress and coping emphasizes that individuals appraise stressors and mobilize coping strategies to restore equilibrium. Coping may be problem-focused (e.g., acquiring emergency kits to increase preparedness) or emotion-focused (e.g., purchasing comfort goods to regulate affect). Prior research has shown that disaster-induced PTSD symptoms can impair adaptive coping, leading to reliance on immediate behavioral strategies such as compulsive or impulsive consumption (22–24). In this study, consumer behavior is conceptualized as a coping mechanism embedded within broader health-related responses, illustrating how psychological distress spills over into everyday decision-making with implications for well-being.

### 2.3 Conservation of resources theory

COR theory (18, 25) posits that stress arises from the loss or threatened loss of resources, including objects, conditions, personal characteristics, and energy. Disasters deplete key resources such as safety, predictability, and meaning, motivating individuals to conserve what remains and invest in substitutes. Post-disaster consumption behaviors, such as redundant stockpiling, insurance uptake, or purposeful purchases aligned with personal values, can be interpreted as strategies for resource protection and restoration (26). This aligns with evidence that individuals with high IUS or heightened death anxiety prefer familiar, low-risk goods that reinforce a sense of control (27, 28).

## 3 Hypotheses development

To provide a coherent structure for the hypotheses, we draw on an overarching guiding theoretical logic. Guided primarily by TMT, we organize our hypotheses around the core proposition that disaster-related mortality salience and trauma-linked distress motivate defensive coping that can manifest in consumption. Within this overarching TMT lens, SCT and CRT specify the key psychosocial mechanisms through which PTSD symptoms translate into post-disaster consumption: (i) DA captures mortality-related affect and existential threat; (ii) IUS reflects uncertainty-driven control restoration and risk-averse coping; and (iii) MLQ-S reflects meaning-making and identity-consistent coping. Accordingly, we present hypotheses in three blocks: (a) the direct association between PTSD symptoms and post-disaster consumption (H1), (b) the three mediator pathways (H2–H5; H9–H11), and (c) perceived media pressure as a boundary condition that moderates PTSD-to-mediator links (H6–H8).

### 3.1 Direct relations

PTSD symptomatology (e.g., intrusions, hyperarousal, avoidance) can spill over into health-relevant coping in everyday life. Under elevated distress, individuals sometimes resort to compulsive or impulsive buying as a short-term affect regulation strategy and a means of perceived control (22, 24, 29). Such purchases can provide temporary relief from anxiety (8). Fear, a typical response to traumatic threat, may shift spending toward utilitarian preparedness goods as a proximal defense, while hedonic, low-guilt treats may function as emotion-focused coping (29–31). A recent meta-analysis confirms that mortality salience systematically shapes consumer responses, reinforcing the expectation that trauma-linked anxiety can lead to both protective and self-soothing purchases (32). From a public health perspective, these behaviors matter because they affect preparedness and psychosocial well-being in disaster contexts (e.g., disaster mental health pathways and resilience). Population studies likewise highlight preparedness behaviors, such as assembling emergency kits or insurance uptake, as health-relevant coping (33).

*H1*. After an earthquake, PTSD symptoms positively influence the purchase of necessities, non-necessities, and self-justification as part of CBDD.

Trauma frequently activates the search for meaning, a process aimed at re-establishing coherence, purpose, and significance. Prolonged searching without resolution can burden well-being (34–36). Recent work demonstrates that brief, meaning-focused interventions can reduce state anxiety following stress, underscoring the health relevance of meaning processes (37). Earthquake-focused studies also document intensified efforts to make meaning that link existential concerns to daily adjustments, such as safety and identity repair (38). Consumer choices may thus operate as purpose-aligned coping mechanisms: spending on sustainable goods, local support, or self-development to restore coherence and psychosocial well-being (39, 40). This aligns with mortality-salience accounts, which show that existential threat reshapes preferences and symbolic investments (5, 32).

*H2a*. PTSD symptoms have a significant and positive impact on MLQ-S.

*H3.* After an earthquake, the search for meaning has a positive influence on the purchase of necessities, non-necessities, and self-justification as part of CBDD.

IU refers to the difficulty individuals experience when coping with ambiguous or unpredictable situations. It is recognized as a transdiagnostic vulnerability linked to emotion regulation deficits and is consistently associated with PTSD after trauma (41, 42). Disaster-exposed populations with elevated PTSD symptoms show greater IU, which predicts anxiety, avoidance, and impaired psychosocial functioning (43–45). Individuals with high IU adopt risk-averse strategies, preferring familiar and predictable options over novel ones (46, 47). In consumer settings, this leads to a reliance on trusted products and a avoidance of uncertainty. Recent evidence shows that individuals with high PTSD severity prefer safe outcomes over risky ones (48), and earthquake survivors with greater IU often engage in resource-protective behaviors such as stockpiling essentials and scrutinizing reliability (49). From a public health perspective, this illustrates how IU channels trauma into coping strategies that both preserve wellbeing and risk over-accumulation.

*H2b*. PTSD symptoms have a significant and positive impact on IU.

*H4.* After an earthquake, IU has a positive influence on the purchase of necessities, non-necessities, and self-justification as part of CBDD.

PTSD can also intensify DA, a transdiagnostic driver of psychopathology and impaired functioning (50). Recent reviews confirm DA’s strong link with anxiety and trauma outcomes, and higher DA is associated with greater odds of probable PTSD in population samples, highlighting its public health relevance (51). Within TMT, mortality reminders prompt defensive behaviors that reduce existential fear. In consumer contexts, this may involve protective purchasing of essentials, hedonic purchases for affect regulation, or symbolic and prosocial choices for identity repair. A recent meta-analysis consolidates mortality-salience effects on consumer responses, while new studies demonstrate that exposure to death-related cues can increase food consumption or shift intentions toward prosocial and environmentally friendly actions (52). Evidence from the 2023 Türkiye earthquakes indicates that DA remains salient among survivors and is associated with lower life satisfaction (53). Interventions can mitigate these effects: mindfulness has been shown to reduce panic buying indirectly by lowering social alienation and DA (54). DA does not always heighten indulgence; it can also encourage saving, insurance uptake, and essential-first spending, which restores perceived control (55).

*H2c*. PTSD symptoms have a significant and positive impact on DA.

*H5.* After an earthquake DA positively influences the purchase of necessities, non-necessities, and self-justification as part of CBDD.

### 3.2 Moderation and mediation relations

ME to traumatic events (earthquakes, terrorist attacks) reliably elicits cognitive–affective reactions and can foster media-induced secondary trauma even among indirectly exposed individuals (56, 57). Exposure is not neutral: disaster content is associated with higher anxiety and acute stress (58), and prior trauma predicts heavier news monitoring that may exacerbate PTSD symptoms (59). In the 2023 Kahramanmaraş context, PTSD has been observed not only among survivors but also among those indirectly exposed via media (5). Journalists covering trauma likewise report symptom escalation with increasing story load and develop coping routines of their own (60). However, sustained and repetitive exposure may also produce desensitization/compassion fatigue (61, 62), and ready-made narratives can saturate interpretive frames and dampen meaning-making (63). From a public health standpoint, media regulation/curation (selective exposure, muting, reliance on official sources) are therefore modifiable determinants of psychosocial outcomes and everyday coping.

We conceptualize ME as perceived media pressure, capturing the intensity/salience of earthquake-related content that individuals experience in daily life; hypotheses are stated at this perceptual, regulation-sensitive level. Integrating the amplification and saturation logics above, we expect outcome-specific moderation:

*H6.* Higher levels of exposure to earthquake-related media will weaken the relationship between PTSD symptoms and MLQ-S.

*H7.* Higher levels of exposure to earthquake-related media will strengthen the relationship between PTSD symptoms and DA.

*H8.* Higher levels of exposure to earthquake-related media will strengthen the relationship between PTSD symptoms and IU.

Consistent with terror-management, stress-and-coping, and resource-conservation perspectives, PTSD is expected to influence CBDD indirectly through distinct psychosocial routes that map onto health-relevant coping (e.g., restoring safety, affect regulation, and coherence):

*H9*. PTSD symptoms influence CBDD through MLQ-S.

*H10*. PTSD symptoms influence CBDD through DA.

*H11*. PTSD symptoms influence CBDD through IU.

**Figure 1:**
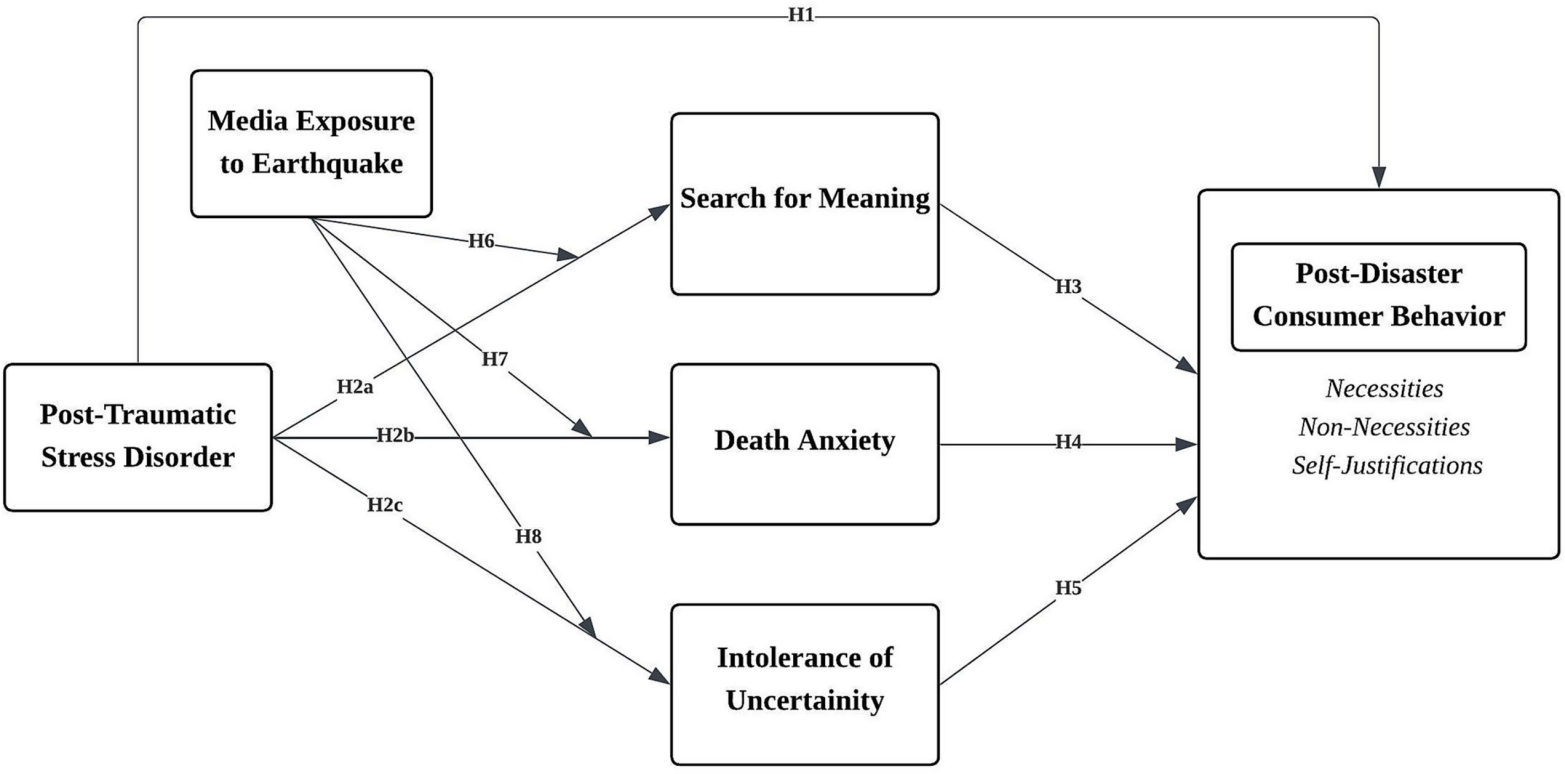
Conceptual model.

Figure 1 depicts the conceptual model based on these hypotheses.

## 4 Quantitative research methodology

The quantitative phase employed partial least squares structural equation modeling (PLS-SEM) to test the hypothesized relationships and assess predictive mechanisms. PLS-SEM was selected for its causal–predictive orientation and capacity to evaluate out-of-sample predictive power (64, 65). Our model includes multiple (serial) mediators and a latent interaction term (ME × PTSD), conditions under which PLS efficiently estimates product-indicator interactions with minimal distributional assumptions (66, 67). Given mild non-normality and a moderate sample (*N* = 350) with reflective constructs, PLS offers stable estimation and prediction-oriented assessment in small-to-medium samples (68, 69). By contrast, covariance-based SEM is optimal when the primary goal is to confirm a strict theory via global fit indices.

PTSD symptoms were assessed with items from a validated PTSD symptom scale that was adapted to the context of the February 2023 earthquakes. The scale comprised 22 items capturing core symptom clusters such as intrusive recollections, avoidance, negative alterations in mood and cognition, and hyperarousal. Respondents indicated how often they had experienced each symptom during the past month on a 5-point Likert scale ranging from (1 = strongly disagree) to (5 = strongly agree), and item scores were averaged to form an overall PTSD symptom index, with higher scores indicating more severe symptomatology. Death anxiety was measured using a standardized 13-item death anxiety questionnaire with the same response format, and higher scores reflected higher levels of anxiety related to death and mortality. Full item wordings and response anchors for both scales are provided in Supplementary Table S1.

### 4.1 Data collection and sampling process

A cross-sectional online survey (Google Forms) was conducted between March and May 2023 to examine the relationship between earthquake-related PTSD symptoms and consumer behavior among adults in Istanbul, Türkiye. A non-probability sample yielded 350 valid responses, all included in the analysis. In line with the study’s focus on tertiary victims, we recruited participants from İstanbul and surrounding areas that were located outside the heavily affected provinces. Respondents were not physically present in the earthquake zone during the February 2023 Kahramanmaraş earthquakes and did not report direct material damage such as injury, property loss, or displacement. Their exposure to the disaster was therefore primarily mediated through news and social media coverage, interpersonal conversations, and institutional communication.

PTSD symptoms were measured with items from the *Impact of Event Scale–Revised* (IES-R) (70). The mediators were MLQ-S, DA, and IUS, each adapted from established scales (71–73). The moderator, in response to perceived media pressure, followed Yeung et al. (74), and CBDD was assessed using an adapted 13-item version of the instrument developed by Di Crosta et al. (29). Three items (DA5, DA8, MLQ9) were reverse-coded to reduce acquiescence bias and identify inconsistent responses. All scales were translated into Turkish and back-translated into English by two independent translators to ensure equivalence. Full item wordings are provided in Supplementary Table S1.

Item-level descriptive statistics for all constructs, including PTSD symptoms and death anxiety, are presented in Supplementary Table S2. Across the 22 PTSD items, mean scores clustered slightly above the midpoint of the 5-point response scale (overall item mean was approximately 3.15), indicating moderate levels of posttraumatic stress symptoms among tertiary victims. For death anxiety, item means were clearly below the midpoint (overall item mean was approximately 2.29), suggesting generally low to mild levels of death-related anxiety, although a subset of respondents still report notable concerns about death and mortality. Sample characteristics are presented in Table 1. Consistent with the study’s focus on indirect exposure, participants were classified as tertiary victims. They were residents of Istanbul who were not directly affected by the February 2023 Kahramanmaraş earthquakes in southern Türkiye but who live under ongoing seismic risk.

**Table 1:** Sample characteristics.

Variables		*n*	%
Gender	Male	167	52.3
	Female	183	47.7
Age	18–25	38	10.9
	26–35	147	42.0
	36–45	78	22.3
	46–55	49	14.0
	56–65	34	9.7
	≥66	4	1.1
Occupation	Student	12	3.4
	Private sector employee	216	61.7
	Civil servant	13	3.7
	Academic staff	43	12.3
	Merchant	2	0.6
	Homemaker	21	6.0
	Small business owners	20	5.7
	Artist (Musician, Painter, Actor, etc.)	1	0.3
	Other	22	6.3
Education	High school and below	56	16.0
	College and equivalent	13	3.7
	Undergraduate	106	30.3
	Master’s degree or above	175	50.0
Income(₺)*	≤8.500	19	5.4
	8.501–15.500	46	13.1
	15.501–22.500	74	21.1
	22.501–29.500	82	23.4
	29.501–37.500	54	15.4
	≥37.501	75	21.4

n: Sample size. ₺ *: The symbol of the Turkish Lira (TL).

### 4.2 Measurement model assessment

The measurement model links latent constructs to their observed indicators. Before conducting the structural analysis, the quality of the scales was evaluated to confirm the factorial structure and psychometric adequacy (75). Following standard practice in confirmatory factor analysis (76), three criteria were assessed: reliability, convergent validity, and discriminant validity.

The model included six latent variables. All factor loadings exceeded the 0.70 threshold except for item PTSD13 (“My feelings about it were kind of numb”), which showed poor loading (0.219) and was removed (77). After deletion, reliability was re-tested. Cronbach’s alpha values for all constructs exceeded 0.70, indicating internal consistency (78).

Convergent validity and discriminant validity were then established. Item properties (loadings, means, SDs, and VIFs) are presented in Supplementary Table S2. Multicollinearity was not a concern, with VIF values ranging from 1.19 to 3.64 (78). Convergent validity was supported by loadings ≥ 0.70 (range 0.703–0.893), composite reliability ≥ 0.80, and AVE ≥ 0.50 across all constructs, consistent with recommended thresholds (79).

Discriminant validity was confirmed. Discriminant validity was confirmed. As shown in Table 2, the square roots of AVE, reported as diagonal values in bold, exceeded the inter-construct correlations (79). Likewise, Table 3 reports that all HTMT values remained below the 0.85 threshold (67).

**Table 2:** Fornell-Larcker criterion.

Construct	Cronbach’s alpha	CR	AVE	CBDD	DA	IUS	ME	MLQ-S	PTSD
CBDD	0.943	0.851	0.717	**0.847**					
DA	0.934	0.907	0.656	0.823	**0.810**				
IUS	0.845	0.829	0.622	0.750	0.772	**0.789**			
ME	0.876	0.801	0.594	0.721	0.689	0.655	**0.771**		
MLQ-S	0.914	0.829	0.571	0.753	0.702	0.640	0.543	**0.756**	
PTSD	0.969	0.872	0.790	0.829	0.808	0.768	0.752	0.743	**0.889**

Bold diagonal values represent the square root of AVE.

**Table 3:** Heterotrait-Monotrait ratio (HTMT) criterion.

Construct	CBDD	DA	IUS	ME	MLQ-S	PTSD	ME x PTSD
CBDD							
DA	0.550						
IUS	0.426	0.459					
ME	0.432	0.317	0.419				
MLQ-S	0.391	0.387	0.242	0.420			
PTSD	0.317	0.201	0.221	0.374	0.612		
ME x PTSD	0.270	0.352	0.147	0.171	0.186	0.382	

The SRMR value was 0.078, which is below the conservative threshold of 0.08 proposed by Hu and Bentler (80). The NFI value was 0.892, indicating an acceptable level of fit and approaching the recommended benchmark of 1 (78). We also report RMStheta = 0.106 (criterion: < 0.12). Finally, the exact fit measures were d_ULS = 0.431 and d_G = 0.218, both of which were below their respective bootstrap-based HI95 reference values (d_ULS HI95 = 0.512; d_G HI95 = 0.264), indicating an acceptable overall model fit.

## 5 Qualitative research methodology

To contextualize the structural results, this section presents five emergent themes, accompanied by representative quotations. Themes were derived from reflexive thematic analysis of 24 Turkish-language interviews, using a hybrid frame anchored in the quantitative model (PTSD → DA/IUS/Meaning—Search (MLQ-S) → CBDD) and the media moderator (PME). For each theme, we provide a brief analytic summary, indicate coverage among participants, and include quotations that illustrate typical patterns as well as negative cases. Quotations are de-identified, translated into English, and back-checked for semantic equivalence; ellipses indicate minor omissions. Where relevant, we reference the corresponding quantitative pathways (*β*, p) and the mixed-methods joint displays (Tables 4, 5) to make integration explicit. The sequence begins with the most prevalent pattern and then moves to mechanisms that qualify or nuance it. Given the cross-sectional survey design and the explanatory purpose of the qualitative phase, the qualitative themes are used to contextualize and elaborate observed relationships; they should be interpreted as associative and mechanism-oriented accounts rather than evidence of causal effects.

**Table 4:** Joint display of core mediation pathways (QUAN ↔ QUAL).

Quant. path (H) and sign	β	Qual. theme → codes	Illustrative quote (ID)	Integrated inference
PTSD → CBDD (H1)	0.496	T3 Little Luxuries, Big Relief→C3.1–C3.7	“I upgraded my headphones… calm is priceless.” (P18)	Beyond mediators, PTSD relates to more (also hedonic) purchasing framed as self-care.
PTSD → MLQ-S (H2a)	0.355	T5 Meaning-Making Through Purposeful Consumption→C5.1–C5.6	“I signed up for a mindfulness course… building something inside me.” (P06)	PTSD raises meaning search; spending shifts to value-congruent, identity-repair purchases.
PTSD → DA (H2b)	0.176	T2 Preparedness as Emotional Insurance→C2.1–C2.7	“I had three flashlights… bought two more; it made me feel I could handle anything.” (P05)	Mortality salience fuels redundant stockpiling as symbolic safety.
PTSD → IUS (H2c)	0.212	T1 Securing the Basics→ C1.1–C1.7	“Buying more gave me relief… if something happens, I’m ready.” (P07)	Heightened uncertainty intolerance redirects spend to low-risk necessities (“control the controllable”).
MLQ-S → CBDD (H3)	0.157	T5 → C5.1–C5.6	“Buying from local artisans who donate… felt like part of the recovery.” (P23)	Meaning search maps onto purposeful purchases (sustainability, local support, self-development).
DA → CBDD (H4)	0.147	T2 → C2.1–C2.6	“Pantry turned into a mini-market… that sight calmed me.” (P20)	DA links to precautionary/backup buying beyond functional need.
IUS → CBDD (H5)	0.172	T → C1.1–C1.7	“Power banks, canned food, first-aid kits… helps me sleep at night.” (P16)	Risk-averse, familiar goods preferred; necessity stockpiling as agency restoration.

**Table 5:** Joint display for the moderator with meta-inferences (areas of convergence/divergence).

Moderated path	Quant moderation (β, p)	Qual. evidence (theme → code)	Convergence/divergence	Meta-inference (resolution)
ME × PTSD → MLQ-S	−0.187, <0.001	T4 Media as a Double-Edged Sword → C4.2 (“narrowing meaning”)	Convergent (directionally)	Higher perceived media pressure narrows meaning pursuits; individuals shift spending to immediate safety/family, away from long-term self-development.
ME × PTSD → DA	−0.094, 0.011	T4 → C4.1, C4.3, C4.4 (media-induced fear, exhaustion; selective avoidance)	Mixed/apparent divergence	Many narratives describe amplification of fear under heavy media, yet the *measured* moderator is perceived media pressure, which may trigger avoidance/regulation, attenuating the PTSD→DA slope on average. Report this as a measurement-process explanation and note heterogeneity.
ME × PTSD → IUS	−0.091, 0.030	T4 → C4.1, C4.6 (tracking official updates for decisions)	Mixed/apparent divergence	Continuous coverage initially heightens vigilance, but selective filtering (official updates) and coping may dampen sensitivity of IUS to PTSD over time. Acknowledge subgroup patterns in text.

### 5.1 Sampling and data collection

We employed an explanatory sequential mixed-methods design (QUAN → QUAL) to deepen and contextualize survey findings on post-disaster consumption among tertiary victims.

The qualitative sample was drawn via criterion-based purposive sampling from survey respondents who: (i) lived in Istanbul in 2023; (ii) self-identified as tertiary (indirect) victims of the February 2023 earthquakes (not physically present in the affected provinces and not first responders); (iii) were 18 years or older; and (iv) consented to follow-up contact. To increase heterogeneity, we applied maximum-variation sampling across gender, age, income, education, occupation, Impact of Event Scale—Revised (IES-R) tertiles, and levels of earthquake-related media exposure observed in the survey. Sample characteristics of the 24 participants are reported in Table 6.

**Table 6:** Sample characteristics of qualitative participants.

ID	Gender	Age	Education	Occupation	Income (try)	Interview duration (min)
P01	Female	28	Bachelor’s	Marketing specialist	35.000–45.000	58
P02	Male	34	Master’s	Civil engineer	55.000–65.000	62
P03	Female	42	Bachelor’s	Teacher	25.000–35.000	55
P04	Female	31	High School	Retail store manager	20.000–25.000	63
P05	Male	29	Master’s	IT consultant	45.000–55.000	60
P06	Female	36	Bachelor’s	Nurse	30.000–40.000	59
P07	Male	47	Bachelor’s	Logistics coordinator	25.000–35.000	64
P08	Female	39	Master’s	Psychologist	40.000–50.000	56
P09	Female	26	Bachelor’s	Social media manager	28.000–35.000	61
P10	Male	53	High School	Small business owner	35.000–45.000	57
P11	Female	45	Bachelor’s	Accountant	30.000–40.000	62
P12	Male	32	Master’s	Architect	50.000–60.000	60
P13	Female	37	Bachelor’s	HR manager	40.000–50.000	55
P14	Female	41	PhD	University lecturer	55.000–65.000	66
P15	Male	30	Bachelor’s	Journalist	28.000–38.000	58
P16	Female	35	Bachelor’s	Civil servant	25.000–35.000	54
P17	Male	48	Master’s	Financial analyst	50.000–60.000	62
P18	Female	33	Bachelor’s	Graphic designer	30.000–40.000	57
P19	Male	44	Bachelor’s	Operations manager	45.000–55.000	60
P20	Female	29	Master’s	NGO program coordinator	28.000–38.000	59
P21	Female	38	Bachelor’s	Event planner	35.000–45.000	55
P22	Male	40	Bachelor’s	Sales director	60.000–70.000	64
P23	Female	27	Bachelor’s	Customer service supervisor	22.000–30.000	53
P24	Male	50	High school	Self-employed	25.000–35.000	58

We completed 24 in-depth interviews. The target size was set *a priori* using information power (81). Given the focused aim, a theory-informed guide grounded in TMT and related constructs (IUS, DA, meaning in life), and the specificity of the sampling frame, approximately 20–25 interviews were deemed adequate. We monitored saturation during fieldwork; code saturation occurred before interview 20, and meaning saturation by interview 22. Two additional interviews confirmed thematic stability.

We conducted semi-structured, one-to-one interviews face-to-face or via secure video conference, according to the participant’s preference and feasibility. Interviews took place within the first 6 months after the earthquakes, concurrent with the survey window, and lasted a median of 60 min. With permission, all sessions were audio-recorded, and brief field notes were captured to capture contextual cues. All procedures complied with established ethical standards for research involving human participants. Prior to participation, respondents were informed about the study’s purpose, confidentiality safeguards, and their right to withdraw at any time. Interview recordings were stored securely and anonymized during transcription to ensure privacy protection. The study received formal ethics approval from the relevant institutional review board, and all procedures adhered to the principles of the Declaration of Helsinki.

The protocol was mapped to the quantitative constructs: (1) everyday manifestations of PTSD symptoms; (2) DA and mortality salience; (3) IUS and control-restoration strategies; (4) search for meaning and identity work; (5) consumption shifts (necessities vs. non-necessities, saving, self-justification); and (6) the influence of earthquake-related media exposure on emotions and choices. The guide was pilot-tested with two individuals outside the final sample and then refined accordingly. The whole question set, organized under the five emergent qualitative themes, is provided in Supplementary Table S3.

### 5.2 Data analysis

Interviews were transcribed verbatim in Turkish de-identified, and managed in MAXQDA. We conducted reflexive thematic analysis (82, 83) using a hybrid coding frame: deductive codes derived from the conceptual model (PTSD→DA/IU/Meaning → Consumption; media moderation) and inductive codes capturing unanticipated mechanisms (e.g., symbolic preparedness, moral accounting, prosocial spillovers). Two trained researchers double-coded an initial subset to calibrate the codebook; one researcher then coded the whole corpus with scheduled peer debriefs to examine interpretations and finalize themes. Representative quotations were translated into English by a bilingual researcher and back-checked for semantic equivalence.

The 24 in-depth interviews generated a total of 73.011 words of transcribed text. Individual transcripts ranged from 2.500 to 3.800 words (M = 3.041; SD = 410), resulting in a sufficiently rich and diverse corpus for thematic analysis, which ensured analytic depth and data saturation.

To assess inter-coder agreement during calibration, two researchers independently coded 20% of transcripts (*n* = 5) in MAXQDA. Cohen’s *κ* for principal codes ranged from 0.82 to 0.91 (M = 0.87), exceeding the 0.80 threshold commonly interpreted as “almost perfect agreement” (84). Discrepancies were resolved by consensus, after which the refined codebook guided the single coder’s completion of the remaining material, supported by periodic peer debriefs to maintain analytic consistency.

Supplementary Table S4 presents the five overarching themes and their 15 sub-themes, with code definitions, participant frequencies, and illustrative links to the quantitative paths. These sub-themes are further disaggregated into 35 fine-grained codes to capture nuanced variations in consumption-related behaviors.

## 6 Results

### 6.1 Hypothesis testing results

**Figure 2:**
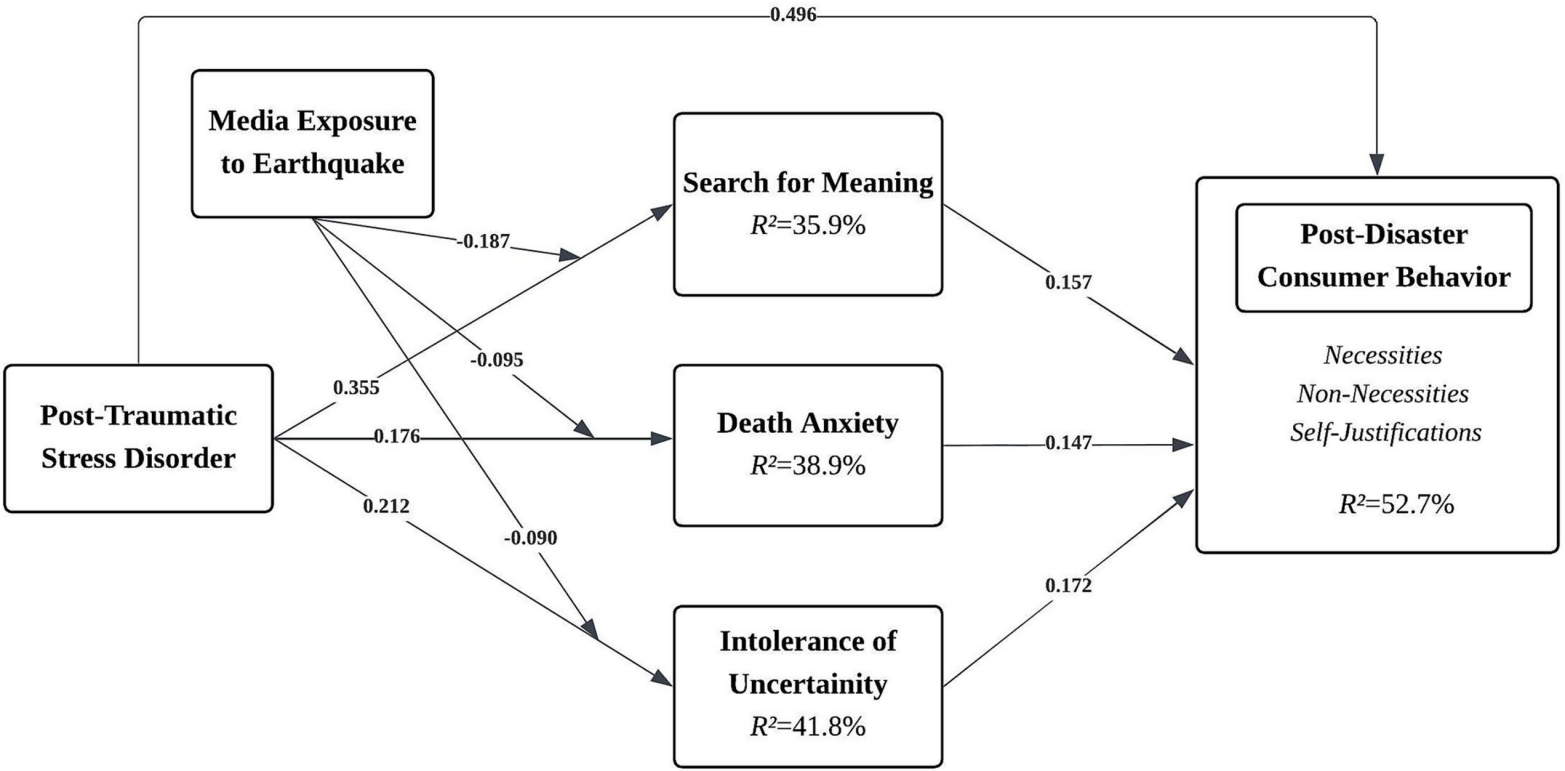
Structural model result.

Following the establishment of the measurement model’s reliability and validity, we proceeded with the structural model evaluation to examine the hypothesized relationships. The analysis was performed using the PLS-SEM algorithm with 10,000 bootstrap resamples, thereby ensuring robust significance testing of direct, indirect, and moderating effects in line with recent methodological recommendations. The structural model with standardized path coefficients and explained variances is illustrated in Figure 2, while the original PLS-SEM output is provided in Supplementary Figure S1.

The results provide strong support for the proposed model. As shown in Table 7, PTSD exerts a substantial direct effect on CBDD (H1, *β* = 0.496, *p* < 0.001) while also demonstrating significant positive influences on MLQ-S (H2a, *β* = 0.355, *p* < 0.001), DA (H2b, *β* = 0.176, *p* < 0.001), and IUS (H2c, *β* = 0.212, p < 0.001). Each of these mediators significantly predicts CBDD (MLQ-S → CBDD: H3, *β* = 0.157, *p* = 0.003; DA → CBDD: H4, *β* = 0.147, *p* = 0.031; IUS → CBDD: H5, *β* = 0.172, *p* = 0.034), thereby underscoring their explanatory relevance.

**Table 7:** Structural model analysis results.

Hypothesis and path	β	SD	2.5–97.5%	t-value	*p*-value	*f* ^2^
H1. PTSD → CBDD	0.496	0.055	(0.388, 0.604)	9.018	**0.000	0.446
H2a. PTSD → MLQ-S	0.355	0.043	(0.271, 0.439)	8.256	**0.000	0.413
H2b. PTSD → DA	0.176	0.038	(0.102, 0.250)	4.632	**0.000	0.222
H2c. PTSD → IUS	0.212	0.035	(0.143, 0.281)	6.057	**0.000	0.385
H3. MLQ-S → CBDD	0.157	0.051	(0.057, 0.257)	3.078	*0.003	0.179
H4. DA → CBDD	0.147	0.068	(0.014, 0.280)	2.162	*0.031	0.092
H5. IUS → CBDD	0.172	0.080	(0.015, 0.329)	2.150	*0.034	0.101
H6. ME x PTSD→MLQ-S	−0.187	0.042	(−0.269, −0.105)	4.452	**0.000	0.235
H7. ME x PTSD→DA	−0.094	0.037	(−0.167, −0.021)	2.541	*0.011	0.117
H8. ME x PTSD → IUS	−0.091	0.042	(−0.173, −0.009)	2.167	*0.030	0.103
H9. PTSD → MLQ-S → CBDD	0.109	0.038	(0.035, 0.183)	2.868	*0.004	–
H10. PTSD → DA → CBDD	0.097	0.046	(0.007, 0.187)	2.109	*0.034	–
H11. PTSD → IUS → CBDD	0.128	0.060	(0.010, 0.246)	2.133	*0.034	–

*p ≤ 0.05; **p ≤ 0.01, R^2^ and Q^2^ values for latent variables: CBDD (R^2^ = 0.527, Q^2^ = 0.512), DA (R^2^ = 0.389, Q^2^ = 0.377), IUS (R^2^ = 0.418, Q^2^ = 0.416), MLQ-S (R^2^ = 359, Q^2^ = 0.348).

The moderating role of ME emerges as negative and significant for all three mediators (ME × PTSD → MLQ-S: H6, *β* = −0.187, *p* < 0.001; ME × PTSD → DA: H7, *β* = −0.094, *p* = 0.011; ME × PTSD → IUS: H8, *β* = −0.091, *p* = 0.030), suggesting that elevated ME systematically attenuates the influence of PTSD on these outcomes.

Effect-size estimates (f^2^; ≈ 0.02 small, 0.15 medium, 0.35 large; 95) show that PTSD → CBDD (f^2^ = 0.446, H1), PTSD → MLQ-S (f^2^ = 0.413, H2a), and PTSD → IUS (f^2^ = 0.385, H2c) are large effects; PTSD → DA (f^2^ = 0.222, H2b) and MLQ-S → CBDD (f^2^ = 0.179, H3) are medium in magnitude; while DA → CBDD (f^2^ = 0.092, H4) and IUS → CBDD (f^2^ = 0.101, H5) are small but meaningful. For the moderator, ME × PTSD → MLQ-S (f^2^ = 0.235, H6) is medium, whereas ME × PTSD → DA (f^2^ = 0.117, H7) and ME × PTSD → IUS (f^2^ = 0.103, H8) are small, consistent with a non-trivial attenuation pattern overall.

The mediation analysis further corroborates the partial mediation mechanism, with significant indirect effects of PTSD on CBDD via MLQ-S (H9, *β* = 0.109, *p* = 0.004), DA (H10, β = 0.097, *p* = 0.034), and IUS (H11, *β* = 0.128, *p* = 0.033). Collectively, the model explains substantial variance in the target construct CBDD (R^2^ = 0.527; Q^2^ = 0.512), while the explanatory power for MLQ-S (R^2^ = 0.359; Q^2^ = 0.348), IUS (R^2^ = 0.418; Q^2^ = 0.416), and DA (R^2^ ≈ 0.039; Q^2^ = 0.377) also meets recommended thresholds for predictive relevance.

### 6.2 Emergent themes and representative quotes

#### 6.2.1 Theme 1. “Securing the basics” consumption as a shield against uncertainty

This was the most prevalent pattern (19 of 24 interviews). Participants across demographics described an almost automatic shift to necessities such as water, canned food, batteries, blankets, and first-aid kits, often beyond their immediate needs. Purchases served psychological preparedness rather than immediate use and were framed as “controlling the controllable” in fragile infrastructures. Even unused items were experienced as calming and protective: “I already had enough canned food, but buying more gave me relief; if something happens, I am ready” (P07). “Two extra power banks I may never use still help me sleep at night” (P16).

A minority viewed this pattern as wasteful or even anxiety-inducing. “Friends bought ten blankets each… I kept thinking, what if nothing happens?” (P03). “Too many supplies just made my home feel like a storage room” (P19). Some reported oscillation, with late-night stocking followed by regret: “I would order at 2 a.m., then wonder in the morning why I did it” (P11).

These narratives align with the quantitative links between PTSD and IUS (*β* = 0.212, *p* < 0.001) and between IUS and CBDD (*β* = 0.172, *p* = 0.034). The necessity purchasing functioned as a stress buffer, restoring perceived safety for many, yet became a visible reminder of vulnerability for some. This is consistent with control-restoration accounts in disaster contexts, where bounded preparedness reduces anxiety, while unchecked accumulation can sustain it and create a household burden, with clear implications for environmental-health risk communication and psychosocial well-being (24).

#### 6.2.2 Theme 2. “Preparedness as emotional insurance” symbolic and redundant stockpiling

This theme appeared in 15 of the 24 interviews and overlaps with Theme 1, but is distinguished by its redundancy. Participants duplicated working supplies to create a symbolic layer of safety rather than to fill actual gaps. Mortality salience was frequently cited as the driver: “I had three flashlights, all working, but bought two more; it made me feel I could handle anything” (P05). “News about water shortages pushed me to order more bottles; my pantry became a mini-market, and the sight calmed me” (P20).

This pattern resonates with TMT, where tangible symbols operate as existential buffers (20). Quantitative results support this interpretation, with PTSD predicting DA (*β* = 0.176, *p* < 0.001), which in turn was associated with both necessity and non-necessity purchases. Some interviewees described redundant buying as an emotional ritual that reassured them regardless of utility: “I check my kit monthly and add items even if not needed; it keeps me sane” (P09).

Counterpoints were present. A subset experienced over-preparedness as an anxiety trap that perpetuated fear rather than easing it: “It became an obsession. My shelves were full, but my mind was not at peace. I realized I was just feeding the fear with more stuff” (P14). Others adopted avoidant strategies to escape constant reminders of risk: “I could buy more supplies, but I choose not to. Seeing them stacked up feels like inviting disaster into my living room” (P22).

Overall, redundant stockpiling served as emotional insurance, bridging DA with preparedness behavior. It provided comfort for many but also introduced clutter, cost and ongoing vigilance for others. This duality highlights adaptive and potentially maladaptive pathways through which environmental-disaster stress is managed, underscoring the value of calibrated preparedness guidance and psychosocial support in public health practice.

#### 6.2.3 Theme 3. “Little luxuries, big relief” hedonic self-care in recovery

This theme was mentioned in 13 of the 24 interviews. Hedonic consumption did not fade after the disaster. Participants reported buying cosmetics, clothing, small electronics, home décor, and gourmet treats to lift their mood, reward endurance, and restore normalcy. What stood out was the lack of guilt. Although prior work links hedonic spending under stress to regret, participants framed these purchases as earned relief and part of recovery.

“After days of watching the news, I bought a silk scarf I had wanted for months. It was not about fashion, it felt like a breath of air” (P12).

“I upgraded my headphones. People might say, why now, but music keeps me calm and calm is priceless” (P18).

Quantitatively, PTSD strongly predicted CBDD (*β* = 0.496), and self-justification emerged as a key cognitive pathway. Narratives demonstrated how reappraisal legitimized small indulgences as low-guilt self-care, supporting emotional regulation and short-term well-being. Still, a minority resisted for financial reasons or out of solidarity with the victims. “I could not buy anything fun, it felt wrong when others lost everything” (P15). Another participant described eventual indulgence as therapeutic. “I finally ordered scented candles, the smell was something other than fear” (P21).

Overall, Theme 3 positions small discretionary purchases as health-relevant coping rather than frivolous spending. For practice, bounded self-care can be normalized in risk communication, while discouraging excessive or debt-creating spending, and aligning environmental-disaster recovery with psychosocial well-being.

#### 6.2.4 Theme 4. “Media as a double-edged sword” exposure, regulation, and coping

This theme appeared in 14 interviews. Continuous exposure to images of collapsed buildings and survivor stories amplified fear, increased DA and IUS, and triggered precautionary purchases. “After every video of collapsed buildings, I ordered more items for my emergency bag” (P10). “Seeing people waiting for aid made me buy thermal clothes and sleeping bags, what if it is me next time” (P17).

At the same time, the statistical model indicated that perceived media pressure attenuated average PTSD translations into DA and IUS (PTSD→DA: *β* = −0.094, PTSD→IUS: *β* = −0.091) and dampened the PTSD→MLQ-S link (*β* = −0.187). Interviews resolve this tension through media regulation. Some participants curated or avoided earthquake content, which reduced spillover and shifted spending away from pure preparedness. “The more I watched, the more I thought nothing else matters but my children. I stopped buying books or courses and focused on safety” (P01). “I muted all earthquake hashtags. After that, I bought a coffee machine. Life has to go on, or the fear wins” (P13). Several described a cycle of doom-scrolling until anxiety peaked, panic buying, then withdrawal to regain balance, followed by renewed exposure days later.

Theme 4, therefore, portrays media as an active force with two pathways. Unregulated monitoring amplifies anxiety and drives protective consumption. Deliberate regulation buffers fear, narrows priorities to immediate safety, and can reopen space for hedonic or value-aligned purchases. The pattern aligns with the model’s negative moderation and underscores media hygiene as a public health lever in environmental disaster contexts, including time limits, source curation, and the avoidance of sensational feeds.

#### 6.2.5 Theme 5. “Meaning-making through purposeful consumption” values-aligned buying for resilience and wellbeing

This theme was mentioned in 11 of the 24 interviews. Some participants addressed post-disaster uncertainty by aligning their purchases with personal values, long-term aims, and identity repair; for these individuals, spending operated as a symbolic investment in resilience, personal growth, and contribution beyond the self.

Participants described choosing ethically produced goods, sustainability-focused brands, and self-development products, such as online courses, therapy sessions, or meditation tools, to restore coherence and agency. This pattern mirrors the quantitative model. PTSD predicted the search for meaning (MLQ-S, *β* = 0.355, *p* < 0.001), and MLQ-S predicted overall post-disaster consumption (CBDD, *β* = 0.157, *p* = 0.003). Meaning-oriented choices were most visible among those who regulated media inputs, for example, by curating sources or muting keywords, which protected attentional bandwidth for values-aligned decisions.

Illustrative accounts emphasized this shift. “Life is fragile; I chose a mindfulness course instead of random stuff, it felt like building inside me” (P06). “I bought from local artisans donating to relief, so every purchase felt like part of recovery” (P23).

Counter-narratives underscored constraints. Some found values-aligned options financially out of reach. “I wanted fair-trade, but essentials left no budget” (P15). Others reported tension between purpose and survival. “I wanted to donate more, but worried we would need that money for food” (P09).

Theme 5 portrays meaning-making as an active, value-congruent process that supports psychosocial well-being, yet is bounded by budget and attention. In environmental-disaster settings, enabling affordable, verified, and sustainable options can channel recovery toward purposeful identity repair, prosocial contribution, and longer-term resilience.

### 6.3 Integration of QUAN–QUAL findings

This study examined how PTSD symptoms shaped post-disaster consumption among tertiary victims of the February 2023 Türkiye earthquakes using an explanatory sequential mixed-methods design. Quantitatively, PTSD had a direct effect on CBDD and indirect effects through MLQ-S, DA, and IUS, while PME attenuated these pathways. The qualitative phase clarified these mechanisms and boundary conditions, illustrating how they were enacted in daily life. Importantly, these meta-inferences reflect explanatory triangulation across methods and do not imply causal directionality.

The joint displays synthesize these dynamics. Table 4 links mediation paths to qualitative themes and quotes, and Table 5 maps the moderator with meta-inferences on convergence and divergence. Together, they show that necessity-driven preparedness, hedonic self-care, and purpose-driven consumption can coexist within the same population, shaped by psychological states and media regulation strategies.

PTSD was positively associated with IUS (*β* = 0.212, *p* < 0.001), and IUS predicted CBDD (*β* = 0.172, *p* = 0.034). Interviews in Theme 1 described “controlling the controllable” through targeted, low-risk necessities such as water, canned food, hygiene items, power banks, and first-aid kits. These accounts position necessity purchasing as agency restoration under uncertainty and explain why IUS relates to familiar, risk-averse choices in practice, consistent with control-restoration logic. PTSD related to higher DA (*β* = 0.176, *p* < 0.001), and DA predicted CBDD (*β* = 0.147, *p* = 0.031). Theme 2 highlighted redundant stockpiling as symbolic safety, aligning with TMT. Ritualized readiness (for example duplicate blankets or extra flashlights) provided perceived protection beyond functional need, linking mortality salience to preparedness behavior.

PTSD also had a strong direct effect on CBDD (*β* = 0.496, *p* < 0.001). Theme 3 showed that small discretionary purchases were reframed as self-care rather than indulgence, with minimal guilt. Cognitive reappraisal and identity-consistent narratives legitimized low-guilt treats as affect regulation, extending work on stress and discretionary spending. PTSD increased the search for meaning (*β* = 0.355, *p* < 0.001), which in turn predicted CBDD (*β* = 0.157, *p* = 0.003), yielding a significant indirect effect (*β* = 0.109, *p* = 0.004). Theme 5 documented values-aligned choices such as sustainable products, local support, and self-development that offered existential anchoring and identity repair, consistent with logotherapy’s emphasis on purposeful action and resilience.

Perceived media pressure reduced average PTSD-to-mediator translations for MLQ-S (*β* = −0.187, *p* < 0.001), DA (*β* = −0.094, *p* = 0.011), and IUS (*β* = −0.091, *p* = 0.030). Theme 4 reconciles this pattern. Unregulated exposure amplified vigilance and anxiety, whereas selective curation and avoidance dampened spillover and redirected attention to immediate safety or everyday normalcy. Because ME was operationalized as perceived pressure, negative mean-level moderation is consistent with active self-regulation in media use rather than a contradiction. For transparency, we report simple-slope plots and regions of significance for PTSD × ME interactions, probe nonlinearity, and estimate subgroup models by media curation and avoidance.

## 7 Discussion

This study demonstrates that PTSD symptoms among tertiary victims of the 2023 Türkiye earthquakes translated into distinct yet interconnected coping logics that were enacted in everyday consumption. Rather than treating consumption as a narrow economic response, our results position it as a psychosocial process that reveals how individuals regulate stress, restore agency, and pursue meaning in the aftermath of environmental disaster (5, 7). Because our data are cross-sectional, we interpret these findings as associations supported by convergent qualitative explanations, not as evidence of causality.

The uncertainty pathway showed that IUS redirected individuals toward low-risk, familiar necessities. Interviews revealed that the targeted stockpiling of water, canned food, or power banks was less about functional need and more about reclaiming control in fragile infrastructures. This pattern highlights how environmental stress is negotiated through tangible acts of preparedness that provide psychological safety, but also how bounded preparedness can shift into overaccumulation, resulting in diminishing returns for well-being. These findings are consistent with prior work linking uncertainty intolerance to cautious, risk-averse decision-making (27, 46), while also extending disaster-preparedness literature by showing how psychosocial stressors translate into concrete household behaviors.

The mortality pathway revealed that death anxiety linked PTSD to redundant stockpiling. Here, duplicate blankets or flashlights functioned as existential buffers, consistent with terror management mechanisms (20). Importantly, these rituals of readiness illustrate how disaster trauma blurs the line between rational preparedness and symbolic insurance. While such practices can temporarily stabilize survivors, they may also sustain vigilance and keep mortality cues ever-present in the domestic space. This finding echoes earlier evidence that mortality salience heightens protective and materialistic responses (85, 86), but our qualitative data in Table 4 show that for some, redundant stockpiling became anxiety-sustaining rather than anxiety-reducing.

The hedonic self-care pathway suggested that discretionary purchases, ranging from small electronics to scented candles, were reinterpreted as essential for emotional stabilization. Contrary to conventional models that associate stress-induced hedonic buying with guilt (87), participants overwhelmingly justified these choices as earned relief. This reframing suggests that cognitive appraisal processes can neutralize anticipated guilt and reclassify indulgence as legitimate self-care. These findings complement earlier work that linked stress to indulgent consumption (24, 30) but diverge by showing how identity-consistent narratives legitimize such spending during recovery (see Table 4).

The meaning-making pathway confirmed that PTSD heightened the search for meaning, which in turn shaped values-aligned consumption. Interviews showed how sustainability, prosocial support, and self-development purchases became tools for existential anchoring and identity repair. This aligns with logotherapy’s proposition that purposeful action fosters resilience (88, 89) and with research on post-traumatic growth (90). Nevertheless, as noted in Table 4, this pathway was bounded by financial constraints and attentional strain, underscoring why meaning-making may be adaptive for some but frustrating for others.

The media pathway revealed the most striking complexity. Quantitatively, perceived media pressure attenuated the translation of PTSD into mediators, while qualitatively, participants described both amplification and regulation. This apparent contradiction resolves when considering media as an active coping arena: unregulated exposure heightened fear and drove precautionary buying, whereas selective curation and avoidance buffered distress, redirected attention to immediate priorities, and even reopened space for hedonic or purposeful choices. This underscores media regulation as a modifiable determinant of psychosocial outcomes in disaster contexts (56, 58). Prior research has often emphasized the media’s amplifying role (59). However, our findings in Table 5 highlight the heterogeneity of media use and its boundary conditions for well-being.

Together, these findings portray post-disaster consumption as a multi-layered coping system. Necessity-driven preparedness, hedonic self-care, and value-aligned choices are not mutually exclusive; they coexist and shift as individuals navigate changing emotional states and environmental cues. The integration of quantitative and qualitative evidence (Tables 4, 5) demonstrates that these behaviors are contingent on PTSD-linked processes and mediated by death anxiety, uncertainty intolerance, and meaning search, with media shaping the strength and direction of these translations. By situating consumer behavior within the domains of psychosocial well-being and disaster health, the study extends beyond prior marketing-centric accounts (10, 12) and contributes to a holistic understanding of how environmental trauma reshapes daily life.

## 8 Theoretical and practical implications

This study shows how PTSD translates into three distinct coping routes: DA, IUS, and search for meaning. Each route produces a different behavioral signature, such as precautionary necessity purchasing, hedonic self-care, or value-aligned spending. These pathways co-exist rather than replace one another, and individuals may shift between them as dominant states change. Mixed-methods integration clarifies mechanisms: stockpiling as a control-restoration mechanism, hedonic purchases as reappraised self-maintenance, and purposeful buying as a mechanism for meaning-making. Significantly, perceived media pressure did not simply amplify distress but often attenuated PTSD effects through selective regulation. Media use, therefore, operates as a boundary condition for trauma responses. These insights extend prior research on mortality salience and defensive consumption (85) by demonstrating both adaptive and maladaptive coping in vicarious trauma contexts.

The findings carry implications for public health communication and humanitarian practice. Both brands and health authorities should provide empathetic, transparent, and consistent crisis communication to prevent fear-driven purchasing (91, 92). Communication that compresses feed intensity and normalizes selective exposure can counteract stress escalation, consistent with evidence on media-induced anxiety (93). Public health agencies and NGOs can promote ethical preparedness with calibrated checklists and affordable kits that restore agency without fueling excess. Retailers and platforms can support recovery by highlighting sustainable and prosocial choices, framing small comforts as responsible self-care while avoiding fear-based cues.

## 9 Managerial implications for retail, e-commerce, and brand management

The findings have clear implications for how retailers, e-commerce platforms, and brands should design interventions in disaster contexts. Mechanism-level evidence shows that PTSD symptoms translate into precautionary, hedonic, and purpose-aligned consumption through DA, IUS, and meaning-seeking. To support adaptive rather than maladaptive responses, retailers can develop calibrated preparedness bundles and rotation reminders that restore agency while minimizing redundant stockpiling. Price-gouging safeguards and inventory caps may further reduce panic accumulation. Equally important is the visibility of sustainable and prosocial options, which can channel recovery toward resilience-building and values-consistent choices. These strategies translate psychological pathways into commercial practices that not only stabilize demand but also protect consumer well-being during periods of environmental stress.

Effective communication is crucial in preventing fear-driven purchasing from spiraling into dysfunction. Brands and platforms should adopt empathetic, transparent, and consistent messaging that aligns with official risk communication, echoing evidence that a supportive tone and timely acknowledgment stabilize stakeholder emotions (94). Integrating contextual nudges, such as earthquake insurance (DASK) prompts or verified donation toggles, embeds preparedness and prosocial action into everyday purchasing journeys. Anchoring product claims in third-party certifications and linking durability to daily continuity reinforce trust and meaning-seeking motives, while avoiding alarmist framings that amplify anxiety. These implications demonstrate that responsible preparedness requires balancing commercial design with psychosocial considerations, ensuring that recovery is steered toward control restoration, reduced waste, and sustainable well-being.

## 10 Limitations

The findings should be interpreted within the specific context of tertiary victims in an urban, media-dense environment after the 2023 Türkiye earthquakes. Cultural norms of solidarity, preparedness, and consumption morality likely shaped how participants framed acceptable responses. This constitutes a boundary condition rather than a flaw, as the study aimed to capture ripple effects beyond the epicenter where symbolic threat and mediated exposure dominate.

A further limitation concerns the granularity of our exposure data. We did not collect systematic information on whether respondents had close relatives or friends living in the disaster region, nor on their history of prior disaster exposure. As a result, the levels of PTSD symptoms and death anxiety reported here should be interpreted as reflecting a heterogeneous group of tertiary victims whose indirect exposure may vary in intensity and form. Future research should explicitly differentiate tertiary victims by social proximity to the affected areas and prior trauma history, and examine how these factors shape post-disaster psychological responses and consumption patterns. Psychological constructs were measured using validated self-report instruments and supported by qualitative data; however, perception-based measures remain sensitive to affective framing and recall bias. The media construct reflected perceived pressure rather than objective dosage or content valence, since regulation strategies emerged as theoretically central. In addition, the qualitative phase primarily reflects the experiences of urban participants with relatively stable resources; patterns in rural or resource-constrained settings may differ, relying more heavily on communal or non-monetary coping. Finally, the design was cross-sectional, which restricts causal inference despite triangulation, theoretical grounding, and joint displays that enhance interpretability.

## 11 Conclusion

This study advances understanding of how trauma-related states shape everyday coping behaviors in the wake of environmental disasters. Focusing on tertiary victims of the 2023 Türkiye earthquakes, we show that PTSD symptoms translate into three distinct yet coexisting pathways: death anxiety, IUS, and the search for meaning. Each pathway maps onto different forms of coping behavior, including precautionary necessity purchasing, hedonic self-care, and value-aligned consumption. By integrating structural modeling with qualitative narratives, the study demonstrates that consumption is not merely an economic response but a psychosocial mechanism through which individuals restore agency, regulate their emotions, and construct meaning under collective stress.

The findings refine existing theories by identifying media regulation as a critical boundary condition. Rather than operating solely as an amplifier, perceived media pressure often attenuates distress through selective exposure and curation. This nuance extends terror-management, coping, and resource-conservation perspectives to vicarious-trauma contexts where symbolic threat outweighs direct exposure.

Practically, the results inform public health communication, humanitarian practice, and market design. Calibrated preparedness guidance, empathetic messaging, and the promotion of sustainable and prosocial options can channel recovery toward responsible preparedness and psychosocial resilience. By situating post-disaster consumption within the domains of health and well-being, the study underscores that everyday choices are central to how societies absorb, interpret, and recover from environmental catastrophe.

## Data Availability

The original contributions presented in the study are included in the article/Supplementary material, further inquiries can be directed to the corresponding authors.
