# Supplementary material for: The impact of PTSD symptoms on post-disaster consumption: tertiary victims of the 2023 Kahramanmaraş earthquakes in Türkiye
Source: Front Public Health. 2026 Feb 24;14:1703071. doi: 10.3389/fpubh.2026.1703071 (PMC12973316; doi:10.3389/fpubh.2026.1703071)
Supplement: Supplementary file 1 [file Table_1.docx]

Supplementary Material

# Supplementary Figures

**Figure S1.** Graphical output of factor loadings and path coefficients.


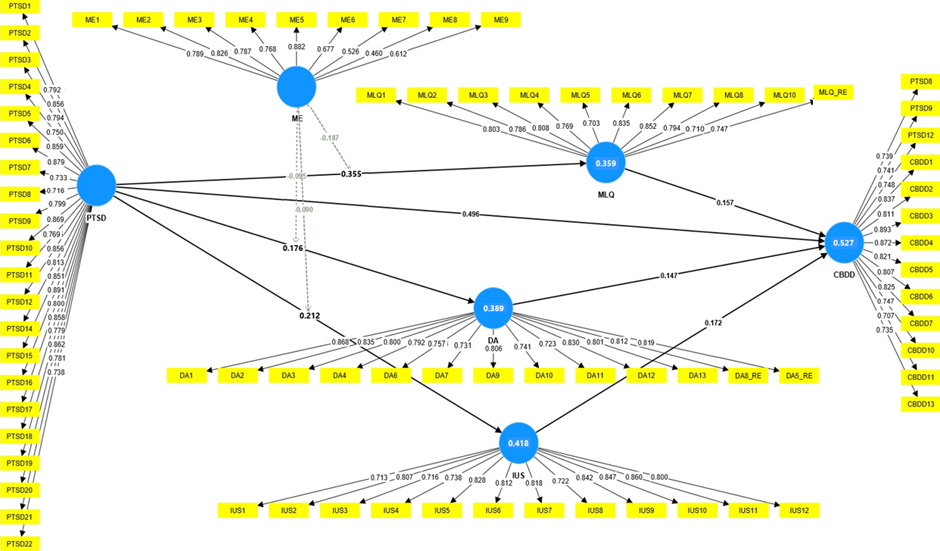


# Supplementary Tables

**Table S1.** Measurement scales and items.

| Consctruct | Items | Reference |
| --- | --- | --- |
| Impact of Event Scale-Revised (IES-R) (Post Traumatic Stress Disorder/ PTSD) | | Adapted from ([70](#Ref70)) |
| PTSD1 | Any reminder of the earthquake keeps my feelings about it alive. |  |
| PTSD2 | Because of the earthquake, I have difficulty staying asleep. |  |
| PTSD3 | Different cues keep making me think about the earthquake. |  |
| PTSD4 | I felt horrified and upset. |  |
| PTSD5 | Whenever I remember or think about it, I cannot manage to keep my emotions under control. |  |
| PTSD6 | I think about the earthquake even when I do not want to. |  |
| PTSD7 | I feel as if the earthquake did not happen or was not real. |  |
| PTSD8 | I avoid reminders of the earthquake. |  |
| PTSD9 | Images related to the earthquake come to my mind. |  |
| PTSD10 | I feel tense and easily startled because of the earthquake. |  |
| PTSD11 | I try not to think about the earthquake. |  |
| PTSD12 | I was aware that I was still very sensitive about the earthquake, but I cannot cope with these feelings. |  |
| PTSD13 | My feelings about the earthquake are losing some of their intensity. |  |
| PTSD14 | I find myself acting or feeling as if I were back at the time of the earthquake disaster. |  |
| PTSD15 | I have difficulty falling asleep. |  |
| PTSD16 | I experience strong emotional ups and downs related to the earthquake. |  |
| PTSD17 | I try to erase the earthquake from my memory. |  |
| PTSD18 | I have trouble concentrating because of the earthquake. |  |
| PTSD19 | Reminders of the earthquake cause me to have physical reactions such as sweating, difficulty breathing, nausea, or heart palpitations. |  |
| PTSD20 | I have dreams about the earthquake. |  |
| PTSD21 | I feel alert and on guard. |  |
| PTSD22 | I try not to talk about the earthquake. |  |
| Perceived Media Exposure (ME) | | Adapted from ([74](#Ref74)) |
| ME1 | I was frequently exposed to media images of collapsed schools or houses |  |
| ME2 | I often encountered media reports about damaged roads, landslides, and ground cracks |  |
| ME3 | Earthquake survivors who were trapped were frequently shown in the media |  |
| ME4 | Seriously injured earthquake survivors were frequently shown in the media |  |
| ME5 | I frequently saw crying earthquake survivors in the media |  |
| ME6 | Injured or frightened children were frequently shown in the media |  |
| ME7 | I frequently saw scenes of people losing family members (death) in the media |  |
| ME8 | I frequently saw close-up footage of corpses in the media |  |
| ME9 | I frequently saw long-distance footage of corpses in the media |  |
| Search for Meaning in Life (MLQ-S) | | Adapted from ([72](#Ref72)) |
| MLQ1 | I understand the meaning of my life |  |
| MLQ2 | I am looking for something that makes my life feel meaningful |  |
| MLQ3 | I always try to find the purpose of my life |  |
| MLQ4 | My life has a clear purpose |  |
| MLQ5 | I have a good idea of what makes my life meaningful |  |
| MLQ6 | After the earthquake, I discovered a satisfying purpose in life |  |
| MLQ7 | I am always searching for something that makes my life feel important |  |
| MLQ8 | I am looking for a purpose or a mission in my life |  |
| MLQ9_RE | My life does not have a clear purpose |  |
| MLQ10 | I am in search of meaning in my life |  |
| Intolerance of Uncertainty (IUS) | | Adapted from ([73](#Ref73)) |
| IUS1 | This unexpected earthquake disaster upset me greatly |  |
| IUS2 | Not having all the information I need about the earthquake upsets me |  |
| IUS3 | One must always look ahead to avoid being caught off guard by the consequences of the earthquake |  |
| IUS4 | An unpredictable small event related to the earthquake can ruin everything despite the best planning |  |
| IUS5 | I always want to know what the future holds for me |  |
| IUS6 | I hate being caught unprepared |  |
| IUS7 | I must be able to organize everything in advance |  |
| IUS8 | When I am not certain, I cannot live life to the fullest |  |
| IUS9 | Uncertainty paralyzes me when it is time to take action |  |
| IUS10 | Because I am not sure about the earthquake, I cannot work very well |  |
| IUS11 | Even the slightest doubt about the earthquake can prevent me from taking action |  |
| IUS12 | I must avoid all uncertain situations related to the earthquake |  |
| Death Anxiety (DA) | | Adapted from ([71](#Ref71)) |
| DA1 | Talking about death makes me anxious |  |
| DA2 | I am very afraid of dying |  |
| DA3 | I am very afraid of dying by being trapped under debris |  |
| DA4 | I am afraid of thinking about having surgery |  |
| DA5_RE | The thought of death does not bother me at all |  |
| DA6 | I am afraid of dying in pain |  |
| DA7 | I am really afraid of having a heart attack |  |
| DA8_RE | I think there is nothing about the future that can frighten me |  |
| DA9 | The sight of a corpse seems terrifying to me |  |
| DA10 | I am often disturbed by how quickly time passes |  |
| DA11 | I often think about how short life really is |  |
| DA12 | The subject of life after death makes me very uncomfortable |  |
| DA13 | I shiver when I hear people talking about strike-slip earthquakes (between magnitude 7 and 8) |  |
| Post Disaster Consumer Behavior (CBDD) | | Adapted from ([29](#Ref29)) |
| CBDD1 | I felt the need to purchase a greater amount of health and safety products (caravan, first aid kit, home insurance, sleeping bag, tent) because of the earthquake |  |
| CBDD2 | I believe I bought the products I needed for the earthquake (flashlight, pocketknife, durable/non-perishable food, water, underwear, soap, etc.) without much thought |  |
| CBDD3 | Compared to before, I felt the need to purchase a greater amount of essential goods |  |
| CBDD4 | Since I did not already own health and safety products, I may spend more time purchasing them |  |
| CBDD5 | I felt the need to buy products that I did not previously need |  |
| CBDD6 | When purchasing non-essential products after the earthquake, I thought about how useful they would be |  |
| CBDD7 | I think about how useful my current non-essential purchases are |  |
| CBDD8 | I did not buy any products after the earthquake that I considered unnecessary |  |
| CBDD9 | Compared to before the earthquake, I felt the need to purchase more non-essential products (e.g., entertainment services, game consoles, luxury goods) |  |
| CBDD10 | Shopping makes me feel better |  |
| CBDD11 | Considering that I will have to live with the reality of earthquakes, I want to enjoy the shopping I do |  |
| CBDD12 | Given that I am saving money for a possible future earthquake, I will still be able to afford new shopping later |  |
| CBDD13 | I am satisfied with my purchases |  |

**Table S2.** Measurement model evaluation results.

| Constructs | Loading | Mean | SD | VIF |
| --- | --- | --- | --- | --- |
| Post Traumatic Stress Disorder (PTSD) | | | | |
| PTSD1 | 0.792 | 3.10 | 1.00 | 1.338 |
| PTSD2 | 0.856 | 3.20 | 1.05 | 2.428 |
| PTSD3 | 0.794 | 3.05 | 0.95 | 2.445 |
| PTSD4 | 0.750 | 3.25 | 1.10 | 1.679 |
| PTSD5 | 0.859 | 3.15 | 1.00 | 1.894 |
| PTSD6 | 0.879 | 3.00 | 1.05 | 1.510 |
| PTSD7 | 0.733 | 3.35 | 1.10 | 2.386 |
| PTSD8 | 0.716 | 3.05 | 0.90 | 1.976 |
| PTSD9 | 0.799 | 3.20 | 1.00 | 2.413 |
| PTSD10 | 0.869 | 3.10 | 0.95 | 1.597 |
| PTSD11 | 0.769 | 3.15 | 1.00 | 1.341 |
| PTSD12 | 0.856 | 3.05 | 0.85 | 1.309 |
| PTSD14 | 0.813 | 3.30 | 1.05 | 1.766 |
| PTSD15 | 0.851 | 3.25 | 1.00 | 2.225 |
| PTSD16 | 0.891 | 3.15 | 1.05 | 1.854 |
| PTSD17 | 0.800 | 3.05 | 1.10 | 2.697 |
| PTSD18 | 0.858 | 3.20 | 0.95 | 1.350 |
| PTSD19 | 0.779 | 3.10 | 1.00 | 1.462 |
| PTSD20 | 0.862 | 3.05 | 1.05 | 1.428 |
| PTSD21 | 0.781 | 3.20 | 1.10 | 2.708 |
| PTSD22 | 0.738 | 3.15 | 1.05 | 2.727 |
| Perceived Media Exposure to Earthquake (ME) | | | | |
| ME1 | 0.782 | 3.42 | 1.08 | 1.522 |
| ME2 | 0.826 | 3.55 | 1.12 | 1.581 |
| ME3 | 0.787 | 3.28 | 1.05 | 2.460 |
| ME4 | 0.768 | 2.97 | 0.98 | 2.065 |
| ME5 | 0.882 | 3.76 | 1.15 | 3.641 |
| ME6 | 0.748 | 3.10 | 0.94 | 1.603 |
| ME7 | 0.766 | 2.84 | 1.02 | 1.456 |
| ME8 | 0.772 | 3.35 | 1.07 | 1.846 |
| ME9 | 0.780 | 3.05 | 0.96 | 1.690 |
| Search for Meaning in Life (MLQ-S) | | | | |
| MLQ1 | 0.803 | 2.68 | 1.05 | 2.684 |
| MLQ2 | 0.786 | 2.73 | 1.02 | 2.729 |
| MLQ3 | 0.808 | 2.78 | 1.07 | 2.777 |
| MLQ4 | 0.769 | 2.67 | 1.00 | 2.674 |
| MLQ5 | 0.703 | 2.15 | 0.95 | 2.151 |
| MLQ6 | 0.835 | 1.95 | 0.88 | 1.945 |
| MLQ7 | 0.852 | 2.83 | 1.08 | 2.829 |
| MLQ8 | 0.794 | 2.86 | 1.12 | 2.862 |
| MLQ9_RE | 0.710 | 1.70 | 0.90 | 1.701 |
| MLQ10 | 0.747 | 1.90 | 0.92 | 1.901 |
| Intolerance of Uncertainty (IUS) | | | | |
| IUS1 | 0.713 | 1.81 | 0.92 | 1.807 |
| IUS2 | 0.807 | 2.82 | 1.10 | 2.816 |
| IUS3 | 0.716 | 1.92 | 0.94 | 1.923 |
| IUS4 | 0.738 | 2.02 | 0.95 | 2.016 |
| IUS5 | 0.828 | 2.81 | 1.12 | 2.812 |
| IUS6 | 0.812 | 2.31 | 1.02 | 2.314 |
| IUS7 | 0.818 | 2.22 | 1.00 | 2.221 |
| IUS8 | 0.722 | 1.82 | 0.90 | 1.824 |
| IUS9 | 0.842 | 2.82 | 1.15 | 2.816 |
| IUS10 | 0.847 | 1.72 | 0.88 | 1.721 |
| IUS11 | 0.860 | 1.22 | 0.85 | 1.224 |
| IUS12 | 0.800 | 2.45 | 1.05 | 2.452 |
| Death Anxiety (DA) | | | | |
| DA1 | 0.868 | 2.05 | 0.95 | 2.054 |
| DA2 | 0.835 | 2.26 | 1.02 | 2.262 |
| DA3 | 0.800 | 2.62 | 1.08 | 2.624 |
| DA4 | 0.792 | 2.74 | 1.10 | 2.735 |
| DA5_RE | 0.819 | 1.96 | 0.92 | 1.964 |
| DA6 | 0.757 | 1.90 | 0.88 | 1.904 |
| DA7 | 0.731 | 2.15 | 0.95 | 2.153 |
| DA8_RE | 0.812 | 2.07 | 0.93 | 2.069 |
| DA9 | 0.806 | 2.87 | 1.15 | 2.869 |
| DA10 | 0.741 | 2.41 | 1.05 | 2.411 |
| DA11 | 0.723 | 2.42 | 1.04 | 2.421 |
| DA12 | 0.830 | 2.24 | 0.99 | 2.241 |
| DA13 | 0.801 | 2.09 | 0.96 | 2.089 |
| Post Disaster Consumer Behavior (CBDD) | | | | |
| CBDD1 | 0.837 | 2.69 | 1.05 | 2.692 |
| CBDD2 | 0.811 | 2.07 | 0.94 | 2.072 |
| CBDD3 | 0.893 | 1.52 | 0.88 | 1.517 |
| CBDD4 | 0.872 | 1.94 | 0.90 | 1.942 |
| CBDD5 | 0.821 | 2.15 | 0.95 | 2.148 |
| CBDD6 | 0.807 | 1.19 | 0.85 | 1.186 |
| CBDD7 | 0.825 | 1.33 | 0.86 | 1.326 |
| CBDD8 | 0.739 | 1.79 | 0.92 | 1.788 |
| CBDD9 | 0.741 | 2.10 | 0.96 | 2.104 |
| CBDD10 | 0.747 | 2.27 | 1.00 | 2.268 |
| CBDD11 | 0.707 | 2.10 | 0.95 | 2.101 |
| CBDD12 | 0.748 | 1.50 | 0.87 | 1.504 |
| CBDD13 | 0.735 | 1.72 | 0.90 | 1.716 |

*RE: Reverse Coded.*

**Table S3.** Semi‑structured interview guide mapped to themes and constructs.

| Theme & Theoretical Target | Primary Question (neutral, time‑anchored) | Suggested Probes / Follow‑ups |
| --- | --- | --- |
| T1 – Securing the Basics *(PTSD → IUS → Necessity purchases; codes C01–C03)* | Thinking about the first six months after the February 2023 earthquakes, did your day‑to‑day “basic preparedness” shopping change in any way? Please walk me through what you did. | Which items came first (water, canned food, hygiene, emergency kit) and why?  When did you start, and for how long did it continue? Any pauses or spikes?  How did these purchases make you feel (safer, more in control)? Any moments of “too much” or “not enough”?  Trade‑offs (postponed other spending, brand switching, bulk vs. small packs).  Information sources (family, experts, official channels, social media) and their influence on timing/choices.  Negative case: If you did not stock up, what held you back (space, budget, skepticism, distrust of advice)? |
| T2 – Preparedness as Emotional Insurance *(PTSD → DA → CBDD; codes C04–C06)* | At any point, did you buy backups/duplicates of items you already had (e.g., extra blanket, power bank, first‑aid)? Tell me about the thinking behind that. | Which duplicates and in what quantities? Practical need vs. “just in case” feeling.  Did having backups reduce anxiety about harm/death or feel like a ritual of readiness?  Storage/rotation routines (expiry checks, drills); any returns or regrets?  Family influence (children/parents/partner) on over‑preparing.  Triggers (aftershocks, TV footage, anniversaries) that restarted buying.  Negative case: If you avoided duplicates, what reassured you instead? |
| T3 – Little Luxuries, Big Relief *(PTSD → CBDD; codes C07–C09)* | Did you make small comfort purchases after the earthquakes (e.g., coffee, chocolate, streaming, spa/massage)? In what ways—if at all—did they help? | Categories and frequency; any budget caps or personal rules.  Any feelings of guilt vs. framing as self‑care (“I needed this to cope”).  Substitution vs. addition (did these replace necessities or sit alongside them?).  Shifts to more affordable treats or, conversely, upgrading quality for comfort.  Role of friends/social media in normalizing or discouraging such purchases.  An instance when a small treat improved your mood—or didn’t.  Negative case: If you avoided comforts, what kept you from it? |
| T4 – Media as a Double‑Edged Sword *(ME × PTSD moderation on DA/IUS/MLQ‑S; codes C10–C12)* | How did earthquake‑related media (TV, Instagram, X/Twitter, Telegram, news apps) affect your feelings and your buying decisions during that period? | Typical daily exposure (times of day, doom‑scrolling moments); perceived pressure vs. merely time spent.  Content that raised anxiety/uncertainty vs. content that felt actionable/empowering; source credibility (official vs. informal).  After specific coverage, did you buy or avoid anything? Concrete examples.  Regulation strategies (muting keywords, curating feeds, following only official updates) and impact on spending.  Did media shift priorities toward immediate family safety/savings and away from longer‑term meaning pursuits?  A turning point when you changed how much you watched and how you shopped.  Negative case: If media had no effect on your shopping, what insulated you? |
| T5 – Meaning‑Making Through Purposeful Consumption *(PTSD → Meaning—Search (MLQ‑S); MLQ‑S → CBDD; codes C13–C15)* | In what ways did the earthquakes influence what matters to you, and how did that show up in your buying or support decisions? | Examples of value‑aligned choices (sustainable goods, buying local, donations/solidarity buying, courses/therapy).  What did these choices represent to you (identity repair, future orientation, community care)?  Trade‑offs (paying more for ethical options, cutting other spending) and how you decided.  Did these purchases help you feel more like yourself or more future‑focused?  Instances where value‑aligned goals conflicted with budget/comfort—how did you resolve it?  Looking ahead, which habits will you keep or drop, and why?  Negative case: If your values didn’t shift, what would your spending have looked like? |

**Table S4.** Full code–theme saturation matrix (n = 24).

| Theme ID | Theme Label | Code ID | Code Description | n* | Quantitative Link (model‑aligned) |
| --- | --- | --- | --- | --- | --- |
| T1 | Securing the Basics | C1.1 | Stockpiling essential goods (water, canned food, batteries) | 19 | PTSD → IUS → CBDD *(qual: necessity stockpiling)* |
|  |  | C1.2 | Emergency kit preparation | 17 | PTSD → IUS → CBDD *(qual: preparedness kits)* |
|  |  | C1.3 | “Control the controllable” mindset | 15 | PTSD → IUS |
|  |  | C1.4 | Prioritizing long‑shelf‑life items | 14 | PTSD → IUS → CBDD |
|  |  | C1.5 | Buying multi‑purpose tools (flashlights, power banks) | 13 | PTSD → IUS → CBDD |
|  |  | C1.6 | Avoidance of discretionary spending under uncertainty | 10 | PTSD → IUS → CBDD *(qual: shift away from discretionary)* |
|  |  | C1.7 | Neighborhood‑level resource pooling | 9 | PTSD → IUS → CBDD *(qual: communal mitigation)* |
| T2 | Preparedness as Emotional Insurance | C2.1 | Redundant stockpiling of already‑owned items | 14 | PTSD → DA → CBDD |
|  |  | C2.2 | Buying high‑utility items “just in case” | 13 | PTSD → DA → CBDD |
|  |  | C2.3 | Symbolic buffering against mortality salience | 12 | PTSD → DA |
|  |  | C2.4 | Backup storage spaces for emergency goods | 11 | PTSD → DA → CBDD |
|  |  | C2.5 | Over‑purchasing food despite adequate stock | 11 | PTSD → DA → CBDD |
|  |  | C2.6 | Increased spending on insurance/safety services | 10 | PTSD → DA → CBDD |
|  |  | C2.7 | Buying for extended family or vulnerable neighbors | 9 | PTSD → DA → CBDD |
| T3 | Little Luxuries, Big Relief | C3.1 | Purchasing small indulgences (coffee, desserts, personal care) | 16 | PTSD → CBDD *(qual: hedonic self‑care)* |
|  |  | C3.2 | Framing hedonic purchases as “self‑care” | 14 | PTSD → CBDD *(qual: cognitive reappraisal)* |
|  |  | C3.3 | Absence of guilt in discretionary spending | 12 | PTSD → CBDD *(qual: guilt suppression)* |
|  |  | C3.4 | Gift‑giving as emotional repair | 11 | PTSD → CBDD |
|  |  | C3.5 | Trying new restaurants or cafés | 10 | PTSD → CBDD |
|  |  | C3.6 | Upgrading personal comfort items (bedding, clothing) | 10 | PTSD → CBDD |
|  |  | C3.7 | Using online shopping as stress relief | 9 | PTSD → CBDD |
| T4 | Media as a Double‑Edged Sword | C4.1 | Media‑induced amplification of precautionary consumption | 15 | ME × PTSD → DA (−); ME × PTSD → IUS (−) *(qual: amplification when unregulated)* |
|  |  | C4.2 | Media narrows perceived meaning to immediate safety/family | 13 | ME × PTSD → Search (MLQ‑S) (−) |
|  |  | C4.3 | Emotional exhaustion from continuous exposure | 10 | ME × PTSD → DA (−) |
|  |  | C4.4 | Selective media avoidance to protect mental health | 9 | ME × PTSD → DA (−) *(qual: regulation attenuates spillover)* |
|  |  | C4.5 | Seeking “hopeful” content (rescue, solidarity) | 9 | ME × PTSD → Search (MLQ‑S) (−) |
|  |  | C4.6 | Tracking official updates for decisions | 8 | ME × PTSD → IUS (−) |
| T5 | Meaning‑Making Through Purposeful Consumption | C5.1 | Purchasing items aligned with personal values | 14 | PTSD → Search (MLQ‑S) → CBDD |
|  |  | C5.2 | Supporting local/small businesses aiding relief | 12 | PTSD → Search (MLQ‑S) → CBDD |
|  |  | C5.3 | Investing in self‑development (books, courses) | 9 | PTSD → Search (MLQ‑S) → CBDD |
|  |  | C5.4 | Donations and charity‑focused buying | 9 | PTSD → Search (MLQ‑S) → CBDD |
|  |  | C5.5 | Choosing sustainable/ethical brands | 8 | PTSD → Search (MLQ‑S) → CBDD |
|  |  | C5.6 | Volunteering‑related purchases (uniforms, supplies) | 8 | PTSD → Search (MLQ‑S) → CBDD |

*n: Indicates the number of distinct participants who mentioned the code at least once.
